# Lectures on the Exploration and Treatment of Diseases of the Chest

**Published:** 1840-06-13

**Authors:** W. W. Gerhard


### MEDICAL EXAMINER.

DEVOTED TO MEDICINE, SURGERY, AND THE COLLATERAL SCIENCES.

No. 24.] PHILADELPHIA, SATURDAY, JUNE 13, 1840. [Vol. III.

### LECTURES ON THE EXPLORATION AND TREATMENT OF DISEASES OF THE CHEST.

BY W. W. GERHARD, M. D.

Lecture V. concluded.—*The Rhonchi.*

There are a set of sounds produced by the respiration in certain states of disease of the chest, which are totally unlike the sounds heard in health. These sounds are called the rhonchi; and they are produced by impediments to motion, either of the lungs themselves, or of the air in the bronchial tubes. Those which belong to the lungs proper are caused by obstacles to the passage of the air through the bronchial tubes; these are the most interesting and important of the class.

There is another set of rhonchi, which arise from the friction of the serous membranes in the chest, and are common to both the lungs and the heart. They occur when the effusions in these membranes consist chiefly of lymph which coats the surface of the serous tissues sufficiently to cause a slight creaking sound. This creaking or friction sound in the pleura, takes place during both inspiration and expiration, but especially at the commencement of the expiration, when the ribs first begin to sink down, and the pleura is drawn rather rapidly over the ribs. It is not limited to a single spot, but shifts about with the dilatation and contraction of the chest; and is generally most evident about the lower angle of the scapula, and often extends from that point across the axilla to the sternum. It is a sign which is proper to pleurisy, either primary or secondary; and it is in general readily recognised after the bronchial rhonchi are known, especially if the friction be sufficient to give to the parietes of the chest a thrilling motion, which may be felt by the hand.

The rhonchi, properly so called, are divided into the moist and the dry. The moist rhonchi are the mucous, including the gurgling of cavities, the sub-crepitant, and the crepitant. The dry rhonchi are the sonorous and the sibilant, to which may be added the dry crepitant.

The *moist rhonchi* are caused by the resistance offered by a liquid in the tubes or vesicles to the passage of the air; the liquid forms bubbles of various sizes, and their successive breaking is the chief cause of the rhonchus. The dry rhonchi are produced by real thickening or spasmodic contraction of the mucous membrane, which gives a musical tone to the respired air; they are most evident in the expiration, while the moist rhonchi are for the most part heard during the inspiration. The rhonchi are not necessarily permanent, except the crepitant rhonchus; for the obstructions forming mucous rhonchus, or the thickening of the larger tubes may be removed for a time, in many cases, by an effort of coughing.

The mucous rhonchus is the loudest of the moist rhonchi; it is caused by the breaking of bubbles of tolerable size contained in the larger tubes; the sound is readily enough recognised, and is scarcely ever mistaken, even on a first examination. This is the sound which is often audible at a little distance from the chest of the patient, especially if it extend over a large portion of the lungs. The mucous rhonchus is heard wherever there is an abundant secretion of liquid into the larger bronchi; now this generally arises from the second stage of bronchitis, but it is quite common in phthisis and the third stage of pneumonia; and the blood which is poured into the bronchi in hæmoptysis, may give rise to the same phenomena. The mucous rhonchus is generally heard both in the inspiration and expiration, as the air returns with sufficient force from the lungs to agitate the liquid, and form bubbles.

There are two varieties of the mucous rhonchus, which are almost peculiar to phthisis; these are the dry crackling, produced by the softening of the thick, pasty matter of tubercle, which gives a peculiarly dry and sharp sound, and the loose, but concentrated gurgling of a cavity. Any disease which gives rise to a cavity in the substance of the lung, will produce this cavernous gurgling; hence it may arise from gangrene of the lungs, pneumonia, or even a dilated bronchus. But as cavities depend much more frequently upon phthisis than any other cause, probably nine-tenths of those which you meet with may be referred to softened tubercles. The gurgling differs from mucous rhonchus merely by its greater concentration; it is in this respect that, like the other signs of cavities, it is distinguished from those of the bronchi; and it passes into mucous rhonchus by an insensible gradation. You may place, therefore, the dividing line between the mucous rhonchus of small cavities, and of the bronchi, where you please. Large cavities can never be mistaken. But there are some cases of dilatation of the bronchial tubes which extend over a considerable portion of the lung, in which the secretion of liquid is abundant, and the mucous rhonchus very similar to that of an ordinary cavity. The liquid gurgling is heard both in the inspiration and expiration, for the air is reflected from the sides of the cavities during expiration, and of course causes an almost continuous rhonchus. You will find that both the crackling and gurgling are liable to disappear, although the cavity remains; for the liquid secretion may be for a time suspended, or the matter may be expectorated, and the walls of the cavity remain dry.

The subcrepitant rhonchus differs from the mucous in two respects: the bubbles are finer, and they break in a more gradual and regular succession. The rhonchus is therefore confined to the smaller tubes, through which the air passes rather slowly, and the bubbles nearly fill up their calibre. It is heard in various parts of the lungs, but much more frequently at their lower and posterior part than elsewhere, for the liquid accumulates there in the smaller tubes more than in any other part. The subcrepitant rhonchus is heard very faintly during the expiration.

The crepitant rhonchus is the most important of the moist rhonchi. It is either fine or coarse, the latter variety differing very slightly from the subcrepitant. When the crepitant is fine, it is pathognomonic of the first stage of pneumonia; and it is then produced in the vesicles of the lung, and perhaps in the small tubes which ramify through the lobules,—but when it is extremely fine, the sound is probably strictly vesicular, and seems to depend upon two causes, the breaking of the minute bubbles of thick mucus, and the dilatation of the thickened and stiffened vesicles. If the crepitus be rather coarse, it seems to arise more from the smaller tubes than from the vesicles, although this is a point which is not susceptible of a rigorous demonstration. A crepitant rhonchus is a sign which is connected with the parenchyma of the lungs, and can never occur in the larger tubes; and it is not produced by other diseases of the parenchyma than pneumonia, because it is only in the latter disease that you will find the thick, viscid secretion, and the stiffened, yet still dilatable condition of the vesicles. The crepitant rhonchus is strictly confined to the inspiration; the air does not pass in the expiration with sufficient rapidity to break the tenacious liquid. The crepitant rhonchus generally forms trains of bubbles, something like the successive explosion of a small train of wet powder; and the sound is compared to various trivial noises, such as the crackling of salt, the rubbing of a lock of hair; but, like all the signs of auscultation, nothing out of the body gives a correct idea of its character. You must, therefore, learn it in patients labouring under pneumonia; and if you have not opportunities for examining cases in connection with persons who are familiar with the physical signs, I would advise you to select a case in which the pneumonia is advanced to the second degree, and the general symptoms of the disease accord with the physical signs. In such cases your diagnosis of the disease may be regarded as quite certain; and you may trace the crepitant rhonchus as it proceeds from the interior of the indurated lung towards the exterior.

There are certain sounds connected with the pleuræ which are similar, as I have already stated, to the moist rhonchi. These are two in number, the friction sound, and the metallic tinkling which is heard generally when the external air communicates with the cavity of the pleura, but is occasionally observed in cases of large cavities in the substance of the lung. The friction sound differs in some cases very slightly from the sub-crepitant, and I have sometimes been puzzled to discriminate between them; of course I do not allude to the well characterized variety, in which there is a thrilling motion extending along the chest, and felt as well as heard, but to those cases in which the friction is very slight. The deposit of lymph is then generally very small, but such is not necessarily the case, for there may be little friction when the effusion is large, especially if the lung be separated from the pleura by serum, which prevents the two surfaces from coming much into contact. The best method of distinguishing the slighter variety is to attend to the manner in which it follows the act of respiration; in the true sub-crepitous rhonchus the bubbles break regularly, and follow the passage of the air; in the slight friction sound there is not this regularity, and its position is never as permanent; there are besides, generally, some collateral circumstances, such as the existence of the sub-crepitant rhonchus in other parts of the lungs, which will aid in distinguishing the two sounds.

The metallic tinkling is a peculiar sound produced by the escape of bubbles of air from beneath a stratum of liquid, situated in a cavity whose walls are firm and elastic. The liquid must occupy only a portion of the cavity, the upper part remaining filled with air. It was supposed that the sound was caused by a drop of liquid which fell from the upper surface of the fluid. Dr. Bigelow, of Boston, suggested the explanation which is now commonly received, that the sound is not caused by the fall of a drop, but by the bursting forth of a bubble of air from beneath the liquid. This is the case, but it is not necessary that the air should be driven forcibly through the bronchial tubes; a very small portion of air contained within the liquid is sufficient to give rise to the tinkling. The sound is called tinkling, because it is somewhat similar to the light tinkle produced by striking with a pin or some other light piece of metal upon a glass vessel. It is always heard in connexion with the amphoric respiration, which depends upon the physical condition necessary to produce it. The sound, therefore, is not of great practical value.

The *dry rhonchi* are the sonorous, sibilant, and the dry or rustling crepitant; the latter of these is of very little value, and hardly differs from the rustling sound of the respiration, to which I have already alluded. They are, for the most part, heard chiefly during the expiration, and are caused by temporary or permanent thickening of portions of the mucous membrane of the larger or smaller tubes. In the large majority of cases they are heard in the earlier stages of bronchitis, before secretion has occurred, or in the chronic stages of this disease in which the secretion is not sufficient to remove the swelling of the membrane. But they may depend upon a purely spasmodic state of the bronchial tubes, for there is no doubt that these tubes are occasionally subject to spasmodic action.

The sonorous rhonchus is generally very loud and well marked; few of you have ever heard it, without recognising it merely from description. It is a loud cooing sound, somewhat similar to that caused by drawing the bow slowly over the bass string of a violin, or to the cooing of pigeons. The sound may be compared most exactly to the note of the violin, but the rhonchus itself is so peculiar from its deep musical tone, and so unlike any other sound heard in the chest, that you will scarcely mistake it. It is most frequent along the upper part of the lungs, both anteriorly and posteriorly, and cannot be produced except in the larger bronchial tubes, for the smaller ones do not yield so deep a note. In acute bronchitis, and even in the chronic cases of this disease, this rhonchus is so fugitive that it sometimes ceases and returns almost with every act of respiration. But you will generally find it in some portion of the lungs, although it may not remain long in a single spot. It is, however, not always so moveable. In the numerous cases of secondary bronchitis which attend the diseases of the lungs and various acute disorders, the sonorous rhonchus is frequent, but it is not found in the most severe and dangerous cases of these disorders, or at least not exclusively. It is in all cases a sign of bronchitis, and when not connected with the moist rhonchi, generally indicates a mild form of the disorder.

The sibilant rhonchus bears the same relation to the smaller tubes, that the sonorous does to the larger; it is a low, whistling sound, heard during the expiration, generally very short and variable in situation. Of course you will find it in those portions of the chest where the bronchi are rather small, and, at the same time, are not subject to congestion or accumulation of secretion,—that is, at the anterior margin of the lungs. The sibilant rhonchus is chiefly heard in the various stages of bronchitis without effusion, especially in the chronic dry catarrh, and the secondary bronchitis of typhoid fever.

Both these dry rhonchi are easily learned from this description alone, for they have a sufficiently close analogy to the sounds which are selected as objects of comparison. Thus the deep bass note and the musical tone are quite characteristic of the sonorous rhonchus, while a whistling and slightly musical sound are equally characteristic of the sibilant. The latter rhonchus is even more moveable than the sonorous, and is extremely irregular in its time of re-appearance.

The mucous, sub-crepitant, sonorous, and sibilant rhonchi are sometimes heard combined together in a variety of chronic catarrh, attended with asthmatic paroxysms; they were then sometimes called by Laennec the "song of all birds,—cantus omnium avium." More frequently, however, you will find two at least of these rhonchi present at the same time, as the sonorous and sibilant, the mucous and the sub-crepitant; a dry may be combined with a moist rhonchus. This depends upon an obvious cause; the various portions of the mucous membrane may be affected to different degrees, and in one part secretion may have commenced, while another remains turgid and dry; besides the secretions tend to accumulate at the posterior and inferior part of the lungs; hence you will find the moist rhonchi sometimes in this position, when the same inflammation gives rise merely to a dry rhonchus elsewhere. The rhonchi may also be connected with other physical signs, as the bronchial respiration and resonance of the voice; and it is sometimes a matter of some difficulty to distinguish them. This is especially the case with the bronchial respiration and the sonorous rhonchus; one not accustomed to these signs may easily mistake one for the other when they occur singly; and if combined, the sonorous rhonchus may mask the bronchial respiration to an inexperienced observer, for both these signs are chiefly heard during the expiration, and there is a certain degree of similarity between them. The only certain distinguishing mark is to examine the part of the chest by percussion; if this be flat it will prove that there is bronchial respiration wherever the tubes are large; if both bronchial respiration and sonorous rhonchus are present at the same time, the flat percussion is so far useful that it indicates the more important sign. The chances of error, therefore, become extremely small, and are still more diminished if you attend to the musical tone in the sonorous rhonchus; this does not characterize the bronchial respiration, which is a pure blowing sound.

After having gone through the description of these sounds you will be tempted to make the same remark which has often been repeated to me. That is, that the difficulty is not in understanding the description of the sounds, but in acquiring the habit of rapidly and readily recognising them. To be practically useful you must distinguish them with certainty, and you must do this without great loss of time to yourself, or the fatigue to your patient which necessarily results from a protracted examination. If you are tempted to lay too much stress upon your newly acquired knowledge, you may perhaps be tempted to fall into the errors against which I have warned you at the beginning of the course, that is, of trusting too much to your physical diagnosis.

Now, you must avoid both these errors, and you will do this by the same means; that is, by making your diagnosis by the general symptoms, and merely adding the physical examination to this as a matter of instruction, until you are sure of your own progress. Those of you who follow my demonstrations will not need this caution, because each step is pointed out, and every part commented upon as it presents itself. The caution is designed for those who trust chiefly to their unaided exertion; these are, under ordinary circumstances, sufficient, though necessarily attended with more trouble, and requiring more time. I shall bear these remarks in mind when describing individual diseases, and will group the physical and general signs together, that one may mutually assist the other.

There are another set of symptoms which are not physical, yet are so local in their character that they should be described before you proceed to the study of special diseases; these are the cough and expectoration, which may properly form the subject of another lecture.

